# Using Structure to Explore the Sequence Alignment Space of Remote Homologs

**DOI:** 10.1371/journal.pcbi.1002175

**Published:** 2011-10-06

**Authors:** Andrew Kuziemko, Barry Honig, Donald Petrey

**Affiliations:** 1 Howard Hughes Medical Institute, Department of Biochemistry and Molecular Biophysics, Columbia University, New York, New York, United States of America; 2 Center for Computational Biology and Bioinformatics, Columbia University, New York, New York, United States of America; University of California San Diego, United States of America

## Abstract

Protein structure modeling by homology requires an accurate sequence alignment between the query protein and its structural template. However, sequence alignment methods based on dynamic programming (DP) are typically unable to generate accurate alignments for remote sequence homologs, thus limiting the applicability of modeling methods. A central problem is that the alignment that is “optimal” in terms of the DP score does not necessarily correspond to the alignment that produces the most accurate structural model. That is, the correct alignment based on structural superposition will generally have a lower score than the optimal alignment obtained from sequence. Variations of the DP algorithm have been developed that generate alternative alignments that are “suboptimal” in terms of the DP score, but these still encounter difficulties in detecting the correct structural alignment. We present here a new alternative sequence alignment method that relies heavily on the structure of the template. By initially aligning the query sequence to individual fragments in secondary structure elements and combining high-scoring fragments that pass basic tests for “modelability”, we can generate accurate alignments within a small ensemble. Our results suggest that the set of sequences that can currently be modeled by homology can be greatly extended.

## Author Summary

It has been suggested that, for nearly every protein sequence, there is already a protein with a similar structure in current protein structure databases. However, with poor or undetectable sequence relationships, it is expected that accurate alignments and models cannot be generated. Here we show that this is not the case, and that whenever structural relationship exists, there are usually local sequence relationships that can be used to generate an accurate alignment, no matter what the global sequence identity. However, this requires an alternative to the traditional dynamic programming algorithm and the consideration of a small ensemble of alignments. We present an algorithm, S4, and demonstrate that it is capable of generating accurate alignments in nearly all cases where a structural relationship exists between two proteins. Our results thus constitute an important advance in the full exploitation of the information in structural databases. That is, the expectation of an accurate alignment suggests that a meaningful model can be generated for nearly every sequence for which a suitable template exists.

## Introduction

Most protein sequences do not have an experimentally determined structure and at least 40% do not even have a sequence homolog with a known structure [1]. Nevertheless, the current Protein Data Bank (PDB) [2] is thought to represent structure space nearly exhaustively [3]–[5]. Therefore, for most proteins, a structural homolog that can serve as a “template” for modeling at least part of its structure is likely to exist. However, the degree of sequence similarity will generally be too low to allow a template to be detected or for an accurate sequence alignment to be found [6]. A central problem is that current alignment methods based on dynamic programming (DP) [7] generate the unique “optimal” alignment (the alignment producing the highest score based on a residue-residue similarity score and a gap penalty), while the “correct” alignment (producing the most accurate model) is not guaranteed to be optimal in terms of this score at low sequence identity ranges.

Numerous variations of both the residue-residue similarity score and gap penalty have been developed to address these issues. Individual residue-based scoring functions have been replaced with more complex profile-profile [8]–[10] and environment-dependent methods [11]–[13]. Recognizing that affine gap penalties typically over-penalize long gaps, several studies have described the probability of a gap as a function of its length or location in the structure with the goal of penalizing it appropriately [14]–[19]. Threading methods [20], [21] incorporate an energy term into the alignment procedure, but they face the drawback of not being compatible with the traditional DP algorithm [22].

Even with these more sophisticated approaches, there are still many issues that will confound the generation of an accurate alignment. Moreover, it is generally necessary to consider an ensemble of alternative alignments in order to produce an accurate model at low sequence identity ranges. Such ensembles are frequently called “suboptimal” since by necessity they have lower scores than the optimal alignment produced by DP. A variety of suboptimal sequence alignment schemes have been reported. Waterman [23] produced an ensemble of alternative alignments by changing the dynamic programming algorithm to return all alignments with scores within a small difference, δ, from that of the optimal alignment. However, the difference between the DP scores of the correct alignment and the optimal sequence alignment can be significant, especially for remote homologues. Increasing δ until it encompasses the correct alignment often produces an unmanageably large ensemble. Keeping δ small returns a more reasonable number, but the alignments tend to deviate negligibly from the optimal alignment.

Saqi and Sternberg [24] adapted this approach to return a more diverse ensemble by penalizing an alignment that is similar to one previously determined. John and Sali [25] used genetic algorithm operators to splice and re-combine alignments in order to achieve the same goal. Chivian and Baker [26] produced alternative alignments by systematically varying the parameters in their optimal alignment method. Each alignment in their returned ensemble was therefore “optimal” (*ie.* highest-scoring) under a different set of conditions. One problem faced by all suboptimal methods is how to adequately sample the gigantic space of possibilities. Jaroszewski *et al* [27] sought to explore the size of alignment space by examining pairs of small and medium-sized proteins (seven or fewer template secondary structures). Even though only “significantly different” alignments were enumerated by disallowing gaps in template secondary structures and ignoring alignment variations in loop regions, tens of millions of alternative alignments were required in some cases to generate the correct one.

We describe here a new method to generate suboptimal alignments, S4 (**S**ampling **S**hifts in **S**econdary **S**tructures), that takes an approach that is fundamentally different from the standard dynamic programming algorithm. In validation tests that we describe below, we show that S4 is highly effective at producing an accurate alignment within a set of 100 top-ranked alternatives and can almost always produce such an alignment within a set of 1000 alternative alignments. The utility of the S4 approach is most evident when the query/template sequence identities are low, but S4 also improves accuracy when the homology is clear. Our results are shown to constitute a significant improvement over DP-based alternative alignment methods, which we show is due to unique features of the algorithm, in particular to the effective use of the 3-dimensional structure of the template. The ability to generate a small set of alignments likely to contain the correct one suggests that S4 offers the possibility of significantly improving the accuracy of homology models, extending the number of sequences that can currently be modeled based on existing structures in the PDB.

## Results

**Figure 1:**
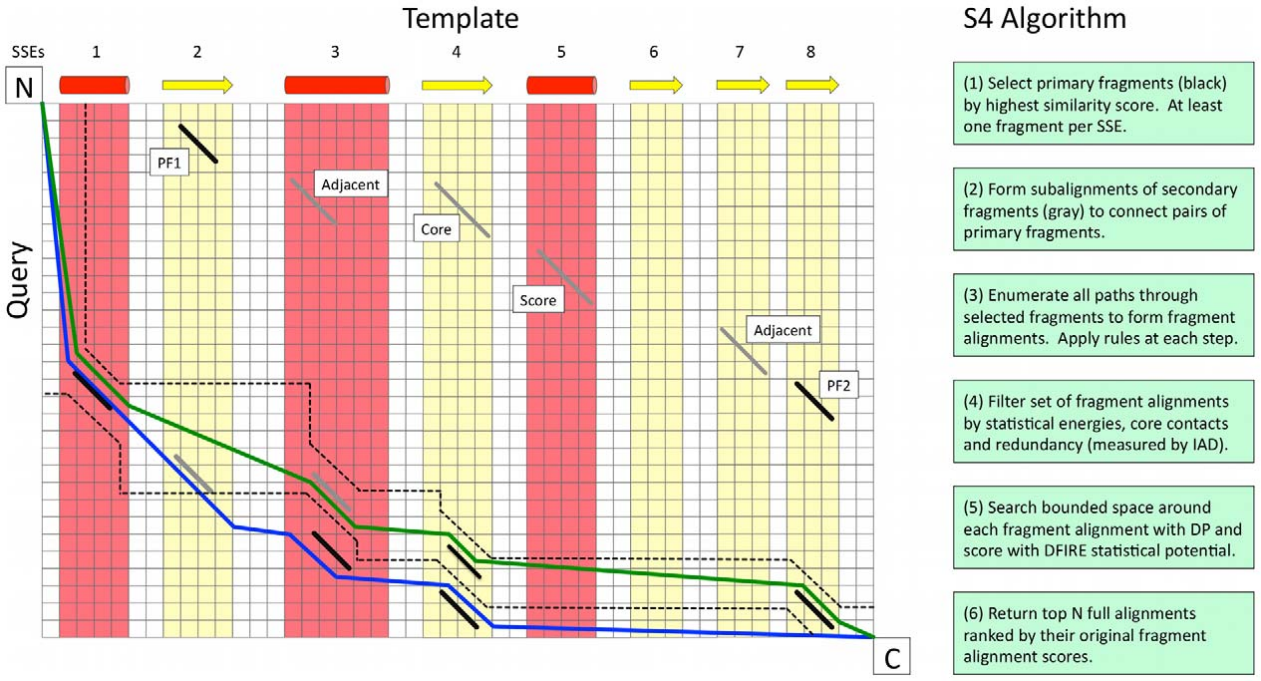
The S4 algorithm. An alignment matrix is depicted with the template sequence and its SSEs on the horizontal axis and the query sequence on the vertical. (1) The algorithm begins by finding high-scoring primary fragments (black, see text for a definition of high-scoring), one primary fragment for each template SSE (not all shown here). (2) To fill in the gaps between primary fragments (such as PF1 and PF2), “secondary” fragments (gray) are identified. Secondary fragments are chosen based on different criteria: if they are in an SSE that neighbors a primary fragment and on a similar diagonal (Adjacent); if they satisfy alignment rules, such as filling a gap in a β-sheet, (Core, see Materials and Methods); or simply being high-scoring (Score). (3) Starting at the N-terminus, the algorithm enumerates all connections to downstream primary and secondary fragments, resulting in a large ensemble of “fragment alignments”. Alignment rules are tested (see Materials and Methods) whenever any fragment is added to an alignment. (4) The number of fragment alignments is reduced by filtering with thresholds based on statistical energies, core contacts and a redundancy measure (see Materials and Methods). (5) To generate a final global alignment from a set of fragments (e.g. the green line, a boundary is defined around each remaining fragment alignment (dashed lines) within which the traditional a DP-based suboptimal algorithm is used to find an ensemble of full alignments. DFIRE then selects the alignment with the lowest/best energy to represent the set of fragments. (6) The process continues until it has returned the top N alignments, ranked by their residue similarity score.

A flowchart for the S4 algorithm is shown in Figure 1. The method starts by searching the DP matrix for a set of short, ungapped alignments bounded by individual template secondary structure elements (SSEs). The rationale is that whatever sequence similarity may exist between query and template will more likely be in SSEs than loop regions. To generate a global alignment, pairs from a high-scoring set of “primary” fragments are connected with lower-scoring “secondary” fragments. This is a crucial feature of S4. In particular, we find that correctly aligned fragments can generally be identified within a very small set of primary fragments, significantly reducing the combinatorial complexity of the alignment problem. This characteristic, combined with the requirement that alignments containing the fragments be structurally plausible (see Materials and Methods), improves accuracy in regions where the relationship between the query and template is less clear. The constraints also allow S4 to remove many alignments from consideration through the application of filters that identify geometrically or energetically unreasonable alignments based on knowledge of the template structure. Filters are also applied to check for redundancy in order to ensure that the alignments represent unique regions of alignment space. (A detailed description of each step of the S4 algorithm and the filters applied is provided in the Materials and Methods.)

### Improvement in alignment accuracy

S4 was tested on a set of target sequences from the CASP [28] experiments (T0129–T0359). Potential templates for each target/query sequence were identified by structurally aligning the native structure to other proteins in the PDB using the ska program [29], [30]. Templates were then selected based on a set of criteria (see Materials and Methods) to ensure that an alignment existed between the template and query structure that would produce a model with a TM-score [31] >0.5, and also that S4 would not be run on sequences longer than 350 residues. The resulting test set contained 3,342 query sequence/template pairs and was heavily populated by those with low sequence identity: over 90% of all pairs had less than 20% identity and more than 60% had less than 10% identity. Overall, there were 137 queries with an average of 24 templates each that satisfied all the criteria. The queries represented at least 65 different SCOP folds (some targets are not classified in SCOP).

We define the correct alignment to be the structure-based sequence alignment between the query and template and evaluate the performance of S4 by comparing it to three DP-based approaches, HMAP [10], hhalign [32], and SP3 [33]. We also compare against a DP-based suboptimal alignment method [23]. We calculate the accuracy of an alignment in different ways. While an alignment algorithm should ideally be able to reproduce the structure-based sequence alignment residue-by-residue, several issues make this an overly sensitive measure of success. For example, consider a situation in which a template contains a helix with an axis that is at an angle with respect to that of the topologically equivalent helix in the query. Because of such differences between the template and query structures, no alignment in this region can be considered strictly correct even though there may be residues in the query and template that occupy roughly equivalent positions in space. The same difficulty occurs in the alignment of loop regions and also in β-strands, where β-bulges can affect alignment accuracy. However, it is clearly desirable for an alignment algorithm to pair residues in topologically equivalent SSEs, even if this pairing does not exactly correspond to the structure-based sequence alignment because of conformational differences.

Because of these issues, we use three measures that reflect a variety of characteristics. We first use a measure called “inter-alignment distance” (IAD). As described in Materials and Methods, IAD corresponds to the average deviation of the position of residues in a given alignment from their correct position in the structure-based alignment. An IAD of 2 implies that, on average, each residue is shifted by two away from its position in the correct alignment, but also implies that topologically equivalent SSEs in the template and query have been correctly paired. Thus IAD is a measure of overall alignment quality. To calculate how well the correct alignment is generated on a residue-by-residue level, we use a measure that we call FDS2, adapted from the FD measure of Sauder *et al.* [34]. This measure is simply the percentage of residues that are within 2 from their position in the correct alignment, with the restriction that this is calculated only in regions corresponding to template SSEs. This restriction results in a more informative alignment metric, since measuring accuracy in the structurally equivalent—but conformationally dissimilar—loop regions of remote homologs imposes a correspondence of residues that is not necessarily meaningful. Finally, to determine whether the models produced from the alignments are actually useful, we directly compare models to the native structure using the TM-score [31].

**Figure 2:**
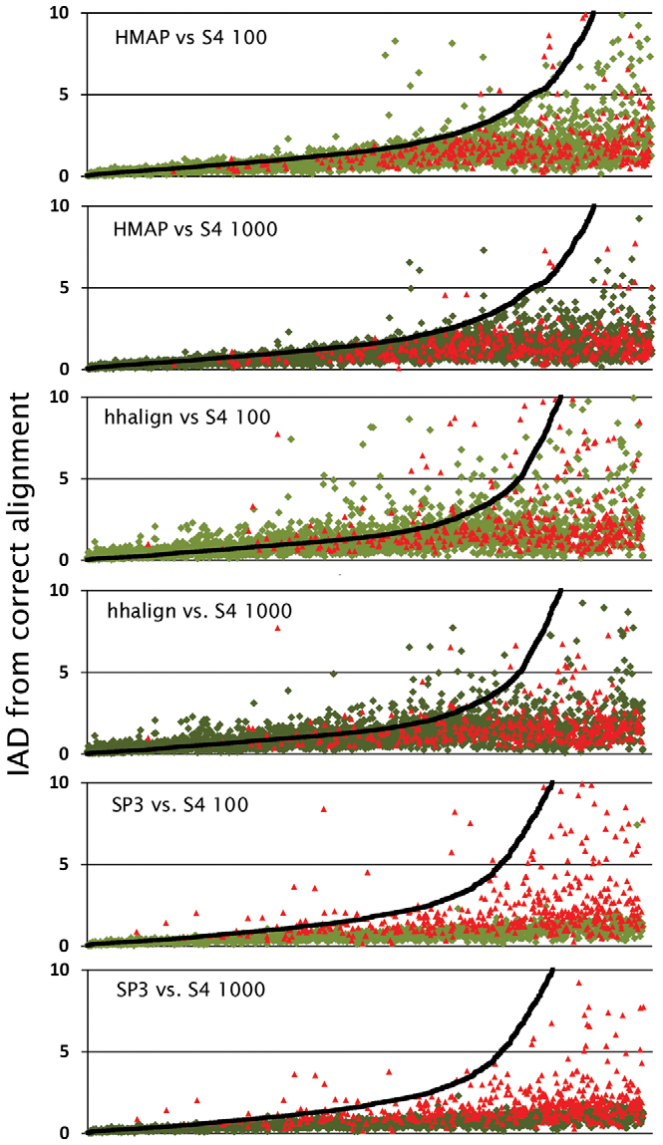
Accuracy of S4 compared to optimal DP-based alignments. For each query/template pair in our benchmark set, we plot two points: one representing the accuracy of the alignment generated by a DP-based method (black squares) and one representing the accuracy of the best alignment from an ensemble generated by S4 (green diamonds and red triangles). Accuracy is calculated using “inter-alignment distance” (IAD, y-axis) from the correct, structure-based sequence alignment and the query/template pairs are ordered along the x-axis according to IAD of the DP-based alignment (lower IAD implies higher accuracy). We take the best S4 alignment from two different ensembles and compare to three DP-based methods indicated in each graph (e.g., the graph labeled “hhalign vs. S4 1000” compares hhalign to the best S4 alignment from an ensemble of 1000 and “SP3 vs. S4 100” compares SP3 to the best S4 alignment from an ensemble of 100, etc.) The green diamonds represent query/template pairs where the template was identified by the DP-based method. Red triangles represent those pairs where the template could only be found by structural comparison to the native structure. Each graph contains data only for those query/template pairs for which an alignment could be generated by the DP-based method (3,343 pairs for HMAP, 2,952 for hhalign and 1,654 for SP3).

Figure 2 plots IAD for the best alignment generated by S4 and the single optimal alignment produced by HMAP, hhalign, and SP3. Points in the figure represent individual query/template pairs and are ordered according to the IAD of the optimal alignment generated by the different methods (i.e., moving left-to-right in the graph corresponds roughly with query/template pairs that range from higher to lower sequence identity). Figure 2 illustrates a central difficulty with most DP-based alignment methods. That is, at the higher range of sequence identities, most methods produce a reasonably accurate alignment, but there appears to be a threshold beyond which an accurate alignment becomes impossible when considering a single, optimal alignment. On the other hand, S4 generates an alignment with improved accuracy at all sequence identity levels and the improvement is quite dramatic at lower identities when the optimal alignment is severely flawed.

Table 1 presents this explicitly, showing IAD values for the different methods averaged over all pairs in several ranges of sequence identity. In the 0–5% identity range, the average IAD for the optimal alignment is over 13, implying that many topologically equivalent SSEs are not correctly paired. In contrast the average IAD for the best S4 alignment found in the top 1000 is 2.3, indicating that S4 is able to find good alignments even in the low identity regime. We note that this is true whether or not the template is identified as a significant hit by the individual methods (E-value<10 for HMAP, E-value<0.001 for hhalign and Z-score<−0.5 for SP3). In Figure 2, the IAD's for the best S4 alignments are colored in light or dark green if the template for that case was identified as significant by the corresponding alignment method, and in red for those templates that are not considered significant.

**Table 1:** Accuracy of S4 alignments at different levels of sequence identity.

		Average IAD		
ID Range (%)	# Pairs	S4 1000	S4 100	HMAP
0–5	567	2.3	4.3	13.6
5–10	1460	1.5	2.3	6.2
10–15	585	0.9	1.0	1.9
15–20	275	0.5	0.5	0.7
20–30	167	0.4	0.4	0.4
30–50	84	0.3	0.3	0.3

ID Range (%)	# Pairs	S4 1000	S4 100	hhalign
0–5	507	2.2	4.1	11.8
5–10	1273	1.5	2.1	6.2
10–15	629	0.9	1.0	2.6
15–20	314	0.5	0.5	1.2
20–30	160	0.3	0.4	0.6
30–50	76	0.3	0.3	0.3

ID Range (%)	# Pairs	S4 1000	S4 100	SP3
0–5	271	2.4	4.8	15.7
5–10	701	1.5	2.3	7.0
10–15	370	0.9	1.1	2.1
15–20	183	0.5	0.5	0.6
20–30	96	0.3	0.3	0.3
30–50	36	0.3	0.3	0.3

The average accuracy of S4 alignments compared to the DP-based optimal alignment programs HMAP, hhalign, and SP3, measured using inter-alignment distance (IAD). The IAD for S4 is based on the best available in an ensemble of either 1,000 or 100 alignments.

Of course, there is an inherent difficulty in comparing the performance of a method which generates an ensemble to a method which generates a single alignment. In fact, the optimal alignment is the most accurate in many cases (about 30% of the time for hhalign and 21% for SP3) and is more often than not in the top 5% in a set of 1,000 alignments ranked by IAD. The average rank is ∼200 however, so there is generally room for improvement, and our main point here is not that the optimal alignment shouldn't be used, but that an ensemble is necessary to generate an alignment that makes an accurate model, especially for highly remote/query template pairs. In practice, the optimal alignment would always be part of such an ensemble.

**Figure 3:**
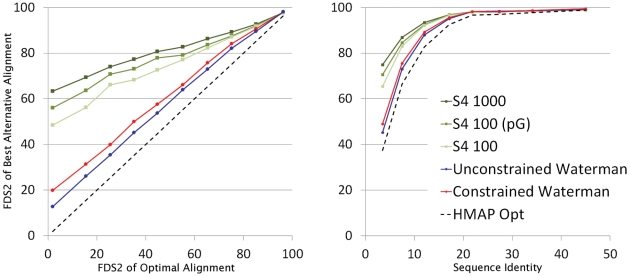
Accuracy of S4 compared to other suboptimal alignment methods. The graphs compare S4 to two versions of a DP-based suboptimal alignment method (see Materials and Methods). In the first panel, the query/template pairs are grouped based on the FDS2 of the optimal alignment (0.0–0.1, 0.1–0.2 … 0.9–1.0), and in the second panel the groupings reflect the sequence identity of the pair (0–5%, 5–10% … 30–40%, 40–50%). In these graphs, a higher FDS2 correlates with a more accurate model. The data points represent the average FDS2 over all pairs in each group, plotted as a function of the IAD of the optimal alignment. The averages are based on the best alignment in the ensemble generated by each method for a query/template pair. For S4, we examined the different ensemble sizes given in the inset legend and used an ensemble of 1,000 for the Waterman based approaches.

To determine the extent to which the improvement in alignment quality of S4 relative to the optimal alignment is due simply to the increased number of alignments generated, we also compared S4 to two versions of the conventional DP-based Waterman algorithm for generating alternative alignments [23], which have been implemented in-house as part of HMAP [10]. Figure 3 shows the results. As discussed above, while the IAD is effective at measuring overall alignment accuracy, it does not define the fraction of residues that are within a specified distance from their position in the correct alignment, and thus in Figure 3 we use the FDS2 measure. We also compare to two versions of the DP-based suboptimal alignment algorithm. A problem with the strict implementation of this algorithm is that alternate alignments can be generated that are not meaningfully different because variations in loop regions produce essentially equivalent models. Thus, we also implemented a modified algorithm which ignores such alignment variations. In the Figure the standard implementation of the algorithm is referred to as “unconstrained Waterman” and the modified version is referred to as “constrained Waterman” (see Materials and Methods and Figure S6 in Text S1 for more detail).

Figure 3 depicts the best FDS2 in the ensemble from each method as a function of the FDS2 of the optimal alignment. The vertical distance above the dotted line represents the improvement over optimal for the best alternative alignment generated. S4 is seen to significantly outperform the DP-based optimal and suboptimal algorithms, particularly when the optimal alignment is flawed. Even the best alignment out of the top 100 S4 alignments is significantly better than the best out of 1000 from the other DP-based methods. A further improvement in accuracy can be obtained by modeling the ensemble of 1000 alignments and using the pG score [35], [36], to select the top 100 alignments based on the quality of the models they produce. Figure 3 also shows the same data as a function of sequence identity. Again, we see that S4 offers a significant improvement compared to all DP-based methods for aligning remote homologs, even when using an ensemble one-tenth as large.

### Evaluation of models from S4 alignments

**Figure 4:**
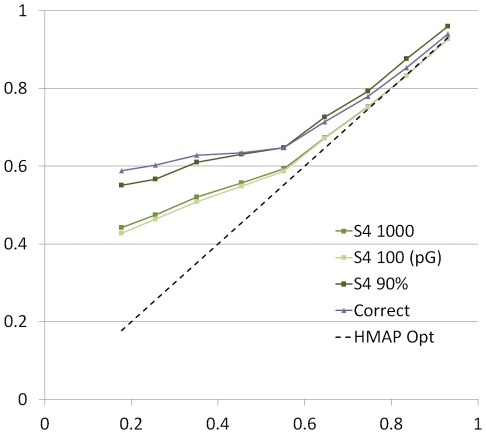
Comparison of model quality. The data set was divided into nine groups based on the quality (as measured by TM-score) of the model from the optimal alignment. The divisions between groups were 0.0–0.2, 0.2–0.3, 0.3–0.4 … 0.9–1.0. The data points represent the average TM-score over all pairs in each group and are plotted as a function of the TM-score of the model based on the optimal alignment. “S4 1000” shows the average TM-score of the best models in the ensemble for each pair. “S4 100 (pG)” is the best out of the 100 models in the ensemble with the highest model evaluation scores. “S4 90%” depicts the 90^th^ percentile of the best S4 models within each group. That is, for 10% of the pairs in each group, S4 produced ensembles in which the best model had a TM-score above the point on “S4 90%”.

The results shown in Figure 2 suggest that S4 generates alignments that are much improved over DP-based optimal methods, but since the IADs of the best S4 alignments are not 0 (i.e., the S4 alignments are not identical to the correct alignment) an important question is whether these improved alignments produce improved 3-dimensional models. To examine this, we made models from the optimal alignment, the correct, structure-based alignment and all alignments in each S4 ensemble for each pair in the data set. The models were then compared to the native structure using the TM-score [31] with results shown in Figure 4. It is evident from the figure that many of the models produced by S4 constitute a significant improvement over the one produced by dynamic programming. The improvement in model quality is most dramatic when the model produced by the optimal alignment is inaccurate. Notably, the best models from S4 are often quite close to the accuracy of the model from the correct alignment. The line labeled “S4 90%” represents the 90^th^ percentile cutoff within each segment, indicating that S4 produced a model for 10% of the pairs that was as accurate as possible, i.e., as good as the model produced by the correct, structure-based alignment.

Figure 4 also shows that evaluating models can significantly reduce the number of models that need to be considered. “S4 100 (pG)” represents the best model of the 100 top-ranked models in the ensemble as determined by the pG score. The proximity of this line to “S4 1000” demonstrates that the pG score consistently ranks the best model from the entire ensemble in the top 100. It is important to be able to reduce the ensemble size in this manner without removing the best models, if further processing of the models is to be carried out (i.e., refinement, minimization, etc.)

### Sampling of alignment space

**Figure 5:**
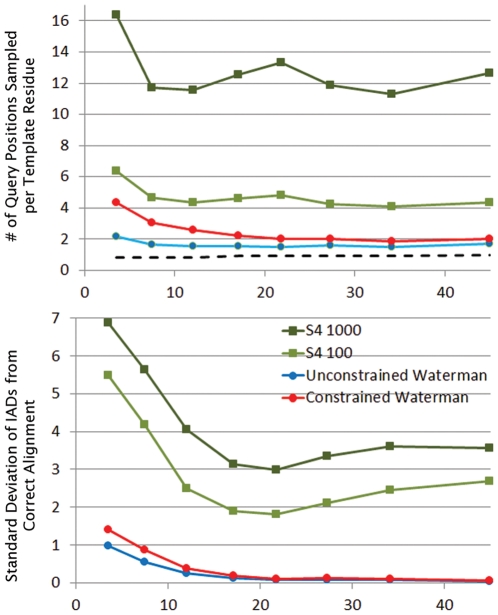
Diversity of alignments in the S4 ensemble. In the top panel we plot on the vertical axis the number of unique query residues sampled at each template residue position in the S4, unconstrained Waterman and constrained Waterman alignment ensembles. For comparison, the optimal alignment sampling, which is necessarily at most one query position per template residue, is also shown. In the bottom panel, we instead plot the standard deviation of the IAD from the correct alignment for each ensemble. A greater standard deviation implies a larger portion of alignment space sampled. In both graphs, the data points represent averages for query/template pairs grouped on the horizontal axis according to sequence identity as in Figure 3. The different ensemble sizes used for S4 are shown in the inset legend and an ensemble size of 1,000 was used for the Waterman based approaches.

Since they use the same scoring function, the improved performance of S4 compared to HMAP seen in Figure 3 is not due to better scoring, but to a broader sampling of alignment space while also avoiding regions that would produce poor alignments. The latter feature is achieved with the rules and filters discussed in Materials and Methods. The ability of S4 to sample broadly should manifest itself in greater sampling at both the residue and whole alignment levels. Indeed, in Figure 5A, we see that S4 samples 3–5 times as many different query residues at each template position compared to the DP-based methods with the same ensemble size.

In Figure 5B, we choose the structure-based sequence alignment as a reference and report the standard deviation of the IAD for all alignments in an ensemble. A low standard deviation indicates that many of the alignments in the ensemble are clustered around a particular distance from the correct alignment, which implies that they are in a narrow region of alignment space. For DP-based methods that region will be centered on the optimal alignment (see Discussion below). We see in Figure 5B that S4 samples broadly within its small ensemble, but can still return an alignment closer to the correct alignment than the DP-based methods (see Figure 3).

## Discussion

**Figure 6:**
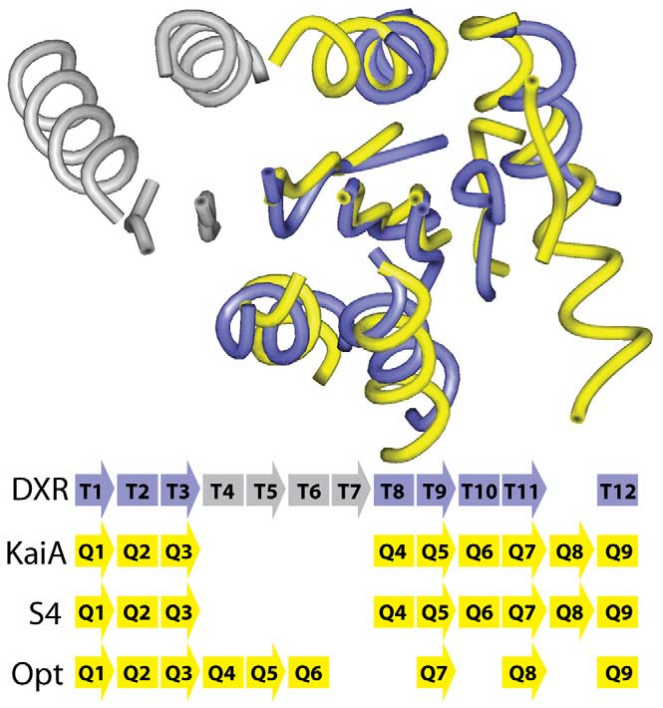
Example of finding the correct alignment between remote homologs. Top: The structural alignment of template DXR (purple) and query KaiA (yellow). Close structural homology clearly exists among the common portion (four structurally equivalent strands and four structurally equivalent helices, loops are not represented for clarity) despite a significant deletion of 4 SSEs in DXR (shown in grey). Bottom: With helices depicted as rectangles and strands as arrows, the top two rows depict the correct correspondence of template and query SSEs based on the structure-based sequence alignment. The next row shows the same correspondence is found in the best alignment in the S4 ensemble. The last line shows the optimal alignment which pairs four SSEs incorrectly.

A specific example illustrates S4's approach to sampling alignment space. Figure 6 depicts a query/template structure alignment along with a listing of their respective SSEs and several ways they are matched in the alignments produced by different methods. The query is the N-terminal domain of KaiA, a non-enzyme circadian clock protein [37] and the template is a single domain of DXR, which is a reductoisomerase [38]. The two proteins are classified as belonging to different folds in SCOP [39] and have less than 2% sequence identity.

Despite being classified as different folds, these two proteins have high overall structural similarity and thus an alignment exists that would generate an accurate model. The structural alignment for this pair describes the proper correspondence of all eight of the SSEs that are common between the template and query, as depicted in the first two rows of the alignment shown in Figure 6. The DP-based optimal alignment contains major flaws and only four out of eight SSEs are in proper correspondence. The poor performance of the DP-based approach is due more to issues with sampling alignment space than to the absence of a detectible sequence relationship between the two proteins. In fact, the eight fragments representing correct correspondences of query and template SSEs were all highly-ranked fragments (or “primary” fragments, in the terminology used in Materials and Methods) as determined by the same HMAP scoring function. All eight correct fragments were chosen within the first 58 (out of a total of 122 used). This local similarity between the profiles is consistent with other local structural, functional and sequence similarities that have recently been described between proteins that have significantly different topologies [40], [41].

Overall, out of an ensemble of 1,000 alignments, the best alignment from S4 has an IAD of 0.56 and an FDS2 of 97% compared to the correct alignment and the TM-score of the corresponding model is 0.50 (compared to a TM-score of 0.57 for the model built from the structure-based alignment). In contrast, the best alignment generated by the constrained Waterman approach (out of an ensemble of 1,000) had an IAD of 15.4. That the improvement in accuracy of S4 is due to differences in sampling can clearly be seen by calculating average IADs of the alignments in each ensemble, but here with respect to the DP-based optimal alignment instead of the correct alignment. The constrained Waterman approach is “trapped” near this incorrect alignment (average IAD of 0.3 and standard deviation of 0.8). Even though S4 samples the DP-based optimal alignment, it also searches far from this alignment (average IAD of 9.4, standard deviation of 4.6, and a maximum IAD of 25.4).

Though we have shown that S4 generates accurate alignments to almost every template appropriate for a given query sequence, we have not discussed how to identify these templates or how to select the correct alignment from the S4 ensemble. However, the results shown in Figure 2 suggest that S4 can be a valuable component of currently used homology modeling strategies. That is, based on the results in Figure 2, most of the appropriate templates that we identify based only on structural similarity to the native structure are recognized as significant using the scoring function associated with the different methods we compare to in the figure. But for a significant majority of these templates an accurate alignment is not possible, at least considering a single alignment generated based on the techniques and information used in the different alignment strategies. This severely limits the number of templates which can be considered useful even if they are recognized.

By building models from templates selected by other methods, but based on alignments generated by S4, these templates can be exploited assuming an accurate model evaluation procedure can be applied. There is a wide array of such tools that range from measures of the suitability for residues to be in a given environment (e.g., Verify3D [42]), to statistical potentials such as D-FIRE, Prosa, or Anolea [43], to all-atom molecular dynamic simulations to estimate the thermodynamic stability of the model (GROMOS [44]). The choice of the best method of evaluation is a complicated one and goes beyond the scope of the current paper where we have focused on S4 as an alignment tool. Nevertheless, for a third of the cases used in our benchmarking, the model with the lowest pG score differs negligibly from the best possible model available from the ensemble (i.e., the best model and the model selected based on pG have TM-scores with respect to the native structure that are within 0.05 of each other). Further, it has been shown that construction of 3D models followed by evaluation using a statistical potential can be used to distinguish true from false homologs when the sequence relationship is ambiguous [35], [45]. These results suggest that more accurate alignments obtained using S4 should significantly expand the number of good templates and models that can be found.

Since S4 produces accurate alignments in nearly every case where there is a structural similarity that leads to an accurate model, this suggests that, using a model-building and evaluation procedure, templates with scores that are outside the range of what is usually considered significant for a particular method could also be identified. Using the widely used tool PSI-BLAST as an example, about half of the templates in our data set were identified as significant (where we define this loosely as E-value<10). As shown in Table 2, in these cases S4 can generate more accurate alignments, in terms of the FDS2 score, than PSI-BLAST. Even for those templates with E-values that are not typically considered useful, (10^−3^<E-value<10), S4 is able to find an alignment that is more than twice as accurate and S4's performance decreases only slightly among the pairs that are not detected at all by PSI-BLAST, which comprise over half the benchmark set. The results shown in Figure 2 indicate that the same conclusion holds no matter what the method used to identify templates. Moreover, preliminary work using a protocol in which templates are selected by PSI-BLAST, models are built from every alignment in the S4 ensemble and evaluated using the pG score as well as other criteria suggests that good templates in this E-value range can be identified with high precision.

**Table 2:** Accuracy of S4 alignments for templates identified by PSI-BLAST.

Psi-Blast E-value	# Pairs	S4 FDS2 (1000)	S4 FDS2 (100)	Psi FDS2
EV<10^−6^	902	95.6	94.9	83.4
10^−6^<EV<10^−3^	164	92.1	91.4	62.1
10^−3^<EV<10	316	88.8	86.1	41.9
No Hit	1516	82.0	76.0	N/A

Alignment pairs have been separated into three regions: clear homology (E-value<10^−6^), intermediate homology (10^−6^<E-value<10^−3^), remote homology (10^−3^<E-value<10) and undetectable homology (template not among PSI-BLAST hits). A default maximum E-value of 10 was used in PSI-BLAST for all queries.

As shown in Figure 5, the primary difference between S4 and other alternative alignment methods is the manner in which alignment space is sampled. The central advantages of S4's sampling are that it generates enough diversity in a small ensemble so that an accurate alignment can be found, while limiting on the number of *potential* alignments that need to be considered (<10 million, see Materials and Methods). In contrast, as we show in Figure 5, DP-based sampling is highly local as a result of the fact that DP must start with the optimal alignment and successively generate other alignments in decreasing order based on their score. This severely limits the amount of diversity that DP can generate and ensures that many more alignments would need to be considered (at least an order of magnitude and probably more) when the DP-based score of the correct alignment is far below the optimal. A low DP score is typical for the more remote query/template pairs in our benchmark, since the correct alignments frequently require long indels or pass through low-scoring regions of the alignment matrix. Moreover, application of the structural filters used in S4 would not be expected to improve this situation, since there are a significant number of inaccurate alignments that satisfy them. Again, if an inaccurate alignment had a better DP-score than the correct one, a DP-generated ensemble would be trapped near the inaccurate alignment, since the local sampling inherent in DP would most likely not generate alignments that break the structural rules in any manageably small ensemble.

While it appears necessary based on our results to consider an ensemble in order to find an accurate alignment, especially for highly remote query/template pairs, it is clearly beneficial to consider the optimal alignment as well. As mentioned above, the optimal DP-based alignment is the most accurate (in an ensemble of 1,000 S4 alignments and 1 optimal alignment) for many cases in our benchmark. An ideal modeling strategy then would be one that generates an ensemble with S4 and simply adds the optimal alignment to that ensemble. This would ensure the best of both worlds at no increase in computational cost. Moreover, the S4 algorithm is independent of the underlying residue-residue scoring function employed. In the work presented here, the HMAP profile-profile method was used, but the sampling algorithm used in S4 could be applied using any other residue-residue scoring function. Therefore, if better scoring functions are available or if future improvements to scoring functions are able to raise the level of accuracy of the DP-based methods, S4's performance using the same scoring function should improve as well.

## Materials and Methods

### Template selection for CASP targets

To ensure that a meaningful structural relationship existed within each query-template pair, several conditions had to be met: the protein structural distance (PSD) [30] could not exceed 0.5 (corresponding to a maximum RMSD of about 3.5 for aligned residues); the sequence identity was less than 50%; and a “pseudomodel” of the query built from the aligned portions of the structure-based sequence alignment and based on the template structure had to return a TM-score [31] of 0.5 or greater compared against the native query structure. A pseudomodel is constructed by simply copying the backbone and Cβ coordinates of residues of the template mutated to the identities of the corresponding aligned residues in the query (unaligned residues are ignored). Also, proteins of length greater than 350 residues were not considered.

### The Algorithm: Overview

The S4 algorithm is described in detail as six distinct steps below (see Figure 1). Overall, the algorithm proceeds as follows. First (Steps 1 and 2), short ungapped alignments entirely contained within template SSEs (‘fragments’) are selected based on their sequence similarity. Any subset of fragments, listed in order from N to C-terminal, is called a fragment alignment. Next, all fragment alignments are exhaustively enumerated and those that pass a set of tests for modelability, are kept. Finally, full alignments are constructed from fragment alignments. The full alignments are generated by standard dynamic programming with the constraint that DP is applied only to a narrow region (defined by the fragment alignment) of the dynamic program scoring matrix. A schematic for the different steps in the process is provided in Text S1, as well as a specific example of how S4's features lead to improvement in alignment accuracy.

### The Algorithm: Selecting fragments (Steps 1 & 2)

Figure 1 shows a typical dynamic programming matrix with the query sequence along the side and the template sequence across the top. The template sequence is divided into columns defined by its secondary structure elements. A diagonal contained within a column is called a “fragment” and represents a short ungapped alignment of the query to the template. To start the alignment process, an initial set of “primary” fragments is identified as follows. Each fragment, (i.e., every diagonal in every column) is examined and is assigned a score that is the sum of the residue-residue similarity scores of the aligned pairs it contains, calculated based on the HMAP profiles [10] of the query and template sequences. The fragment from each column with the highest normalized score (the profile-profile similarity score divided by the length of the fragment) is added to the list of “primary” fragments (black lines in Figure 1). Each template SSE will contain at least one primary fragment and usually several more.

For every pair of primary fragments we perform a recursive search for “secondary” fragments to fill in the region defined by the fragment endpoints, if the fragments in the pair belong to non-consecutive SSE's. For example, in Figure 1, two secondary fragments are chosen for being the highest scoring secondary fragments that are “adjacent” to primary fragments PF1 and PF2. (An adjacent fragment is contained in a neighboring SSE and is on the same or a nearby diagonal.) Other secondary fragments are chosen by virtue of being high-scoring or in an SSE whose deletion would violate the alignment rules (e.g., a missing core strand, see below). This process continues recursively until all regions between non-consecutive fragments in a subset have been filled in.

### The Algorithm: Enumerating and filtering fragment alignments (Steps 3 & 4)

A “fragment alignment” is a list of primary and secondary fragments in order from the N- to C-terminal. Two examples of fragment alignments are shown in Figure 1. The blue and green lines both run alongside two sets of four fragments (which share a common first member). Fragment alignments such as these will later form the basis of full alignments (constructed as described below).

To enumerate all fragment alignments that are possible within our set of primary and secondary fragments, S4 connects the N-terminal pseudo-fragment (upper-left corner of Figure 1) to each downstream primary fragment (either directly or through subalignments of secondary fragments). This process progresses to further downstream fragments until all alignments end at the C-terminal pseudo-fragment (bottom-right corner of Figure 1). After any connection between fragments is established, a set of conditions must be met. If an alignment fails to meet one of these conditions (described below), the enumeration process is discontinued for that particular path. (Some conditions can only be applied when the C-terminal is reached). It should be noted the total number of possible fragment alignment can be calculated efficiently during the above process, and no new fragments are added once the total number of alignments exceeds 10 million.

Some of the conditions placed on the fragment alignments are based on the properties of the alignment itself and some are based on a 3D pseudomodel of the query. The conditions that must be met by each alignment/pseudomodel are described below.

#### Coverage

We are generally not interested in alignments that pair a very small number of residues. Therefore, only alignments where at least 10% of the shorter sequence is aligned to the longer sequence are retained. Since only residues in template SSEs are counted in S4, this fraction represents a somewhat more stringent condition than it may initially appear.

#### Contact order

The contact order for a pseudomodel is defined here as the percentage of its SSE residues containing a Cβ that lie within 6 Å of a Cβ from a residue in a different SSE. Fragment alignments whose pseudomodel has a contact order less than 65% of the contact order of the template itself are rejected. Making this threshold relative to the template ensures that “extended” models will not be built from compact templates, but if the template itself is extended, the fragment alignment will be kept.

#### Strand pairing

There are two general rules governing the pairing of beta strands in homologous proteins that can be used to eliminate bad alignments [46]. First, a paired strand in the template should not become unpaired in the pseudomodel. Second, a core strand of a beta sheet in the template must be present in the pseudomodel if its flanking strands are also present.

#### Loop lengths

Fragment alignments are rejected if there are not enough residues in the query sequence to bridge the gap between any two consecutive fragments. Specifically, we require that 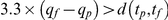, where *q_p_* is the index of the final query residue of the fragment preceding the loop, *q_f_* is the index of the first query residue following the loop, and *d(t_p_,t_f_)* denotes the distance (in Å) in the template structure between the Cα atoms of the corresponding, aligned template residues. The factor of 3.3 was determined by studying a database of several hundred high-resolution structures. It was found that the maximum distance traversed by a loop was slightly over 3 Å per residue, which is, of course, roughly the length of an individual amino acid. We used 3.3 to allow our algorithm to keep some pairings of fragments whose loops would normally be ‘over-stretched’. The purpose of this test was to remove only blatantly incorrect fragment pairings, since loops that were just slightly over-stretched may be fixed when the full alignment is found in Step 5 of the algorithm.

Three other measures were used to eliminate fragment alignments that are unlikely to produce good models: preserved core contacts, query energy and template energy. For an alignment to be kept, all three of these measures must have values above the 66^th^-percentile for each measure and one of these three values had to surpass the 90^th^-percentile. The measures are listed below.

#### Preserved core contacts

A pair of residues in the template structure is considered to be a “core contact” if both residues in the pair are buried (60% or more of surface area inaccessible), have Cβ atoms that are within 6 Å and are both hydrophobic (amino acid types A, F, G, I, L, M, P, W, V and Y). An alignment that pairs hydrophobic amino acids in the query with template residues in a core contact generates a preserved core contact.

#### Statistical energy of query residues

An implementation of the DFIRE statistical potential [47] was used to evaluate each alignment by using the Cα and Cβ positions from the template with the amino acid types of the aligned query residues. A pseudo-Cβ position was determined for glycine residues based on the Cβ position in alanine. Loop residues were not considered in either the calculation of the statistical energies or in the tabulation of inter-residue distances that form the basis of this implementation of DFIRE. The value thus calculated, called the “query energy”, and the proximity of the alignment to the correct one were found to be highly correlated.

#### Statistical energy of template residues

Similar to evaluating the statistical energy of the pseudomodel, we calculate the energy of the aligned template residues, which we term the “template energy”. In effect, this is the statistical energy of a subset of the template structure. The motivation behind this is to recognize and remove alignments that pair query residues with an unlikely combination of template SSEs. This often occurs when the template is a multi-domain protein and the query is a single domain. In these cases, the highest scoring fragments may be spread out across multiple domains of the template in a structure that does not resemble a folded protein. Calculating this value allows S4 to eliminate many such alignments.

#### Redundancy

Lastly, to decrease the redundancy of the final results, some fragment alignments are removed due to their similarity to a higher-scoring alignment. Fragment alignments are considered redundant if they align to the same template SSEs, have all corresponding fragments within a shift of 4, and an inter-alignment distance (IAD) of less than 1.

### The Algorithm: Constructing full alignments (Steps 5 & 6)

At this stage in the process, no full alignments in the normal sense have been created, only fragment alignments, which are just lists of fragments. A round of alignment sampling using the full sequences of the query and template is used to generate a final alignment from each fragment alignment. In this final step, alignments are restricted to a specific region of the dynamic program matrix. The boundaries of the region extend 3 residues above and below the fragments in each fragment alignment. The loop regions are constrained only by the boundaries of the surrounding fragments (dashed lines in Figure 1). Alignment sampling is carried out using the constrained Waterman approach. That is, we apply this algorithm in regions of alignment space that we expect to be unique based on the structure of the template. Again, a pseudomodel is constructed for each alignment which is scored with DFIRE [47] as described above. The alignment with the best/lowest energy is selected to represent the original fragment alignment.

The S4 algorithm typically generates thousands of fragment alignments. A single, full alignment is generated for each one, starting with the highest-scoring, until N unique alignments have been found, where N is the ensemble size chosen by the user. The score of an alignment is simply the sum of the similarity scores of the paired residues in the original fragment alignment minus a flat penalty for each inserted residue. The insertion penalty only applies to residues inserted between template residues and is therefore used to encourage insertions at the termini. Deletions are not penalized since we found that structural considerations enabled us to disallow unreasonable gaps without an explicit penalty. A worked example illustrating each step is provided in Text S1.

### Inter-alignment distance (IAD)

We calculate the distance between any two alignments using a measure similar to the gALD measure developed by Chen and Kihara [48]. If we plot two alignments of the same two sequences on the dynamic programming matrix (Figure 1, blue and green lines) there is a region between them for which we can calculate the area. Dividing this area by the length of the template yields an average height of this region, which can be interpreted as the average distance that a query residue in one alignment is shifted from its position in the other. This average distance we have termed the IAD and it should be considered to have units of residues. This measure is quick to calculate and useful for determining if two alignments occupy the same region of alignment space.

### The standard DP-based alternative alignment methods

The unconstrained Waterman and constrained Waterman in Figures 3 and 5 are implementations of the method described by Waterman. [23]. The “unconstrained Waterman” approach is an unmodified version of that algorithm that that use the HMAP scoring function and gap penalty and generates alternate alignments by allowing the DP procedure to branch to an alternate path at any point in the DP matrix where doing so will lead to an alignment with a score within δ of optimal. However, in the constrained Waterman approach, branching to alternate paths is allowed only when moving between SSE and loop regions (see Figure S6 in Text S1 for more details). For both methods, it is impossible to know which value of δ will generate an ensemble of a desired size. To generate the alignments for comparison, we started with very small values for δ and increased it until the ensemble size exceeded 1000. We then sorted the alignments by their DP-based score and kept only the top 1000.

### Model building and model accuracy

Models were built with the program Nest [29] for all S4 alignments, the optimal HMAP alignment and the correct/structure-based alignment. TM-score [31] was used to evaluate the accuracy of the model compared to the native query structure.

## Supporting Information

Text S1 Supplemental information for “Using structure to explore the sequence alignment space of remote homologs”.
(DOC)
