# Supplementary material for: Using Structure to Explore the Sequence Alignment Space of Remote Homologs
Source: PLoS Comput Biol. 2011 Oct 6;7(10):e1002175. doi: 10.1371/journal.pcbi.1002175 (PMC3188491; doi:10.1371/journal.pcbi.1002175)

**TEXT S1**

**An example of the S4 algorithm**


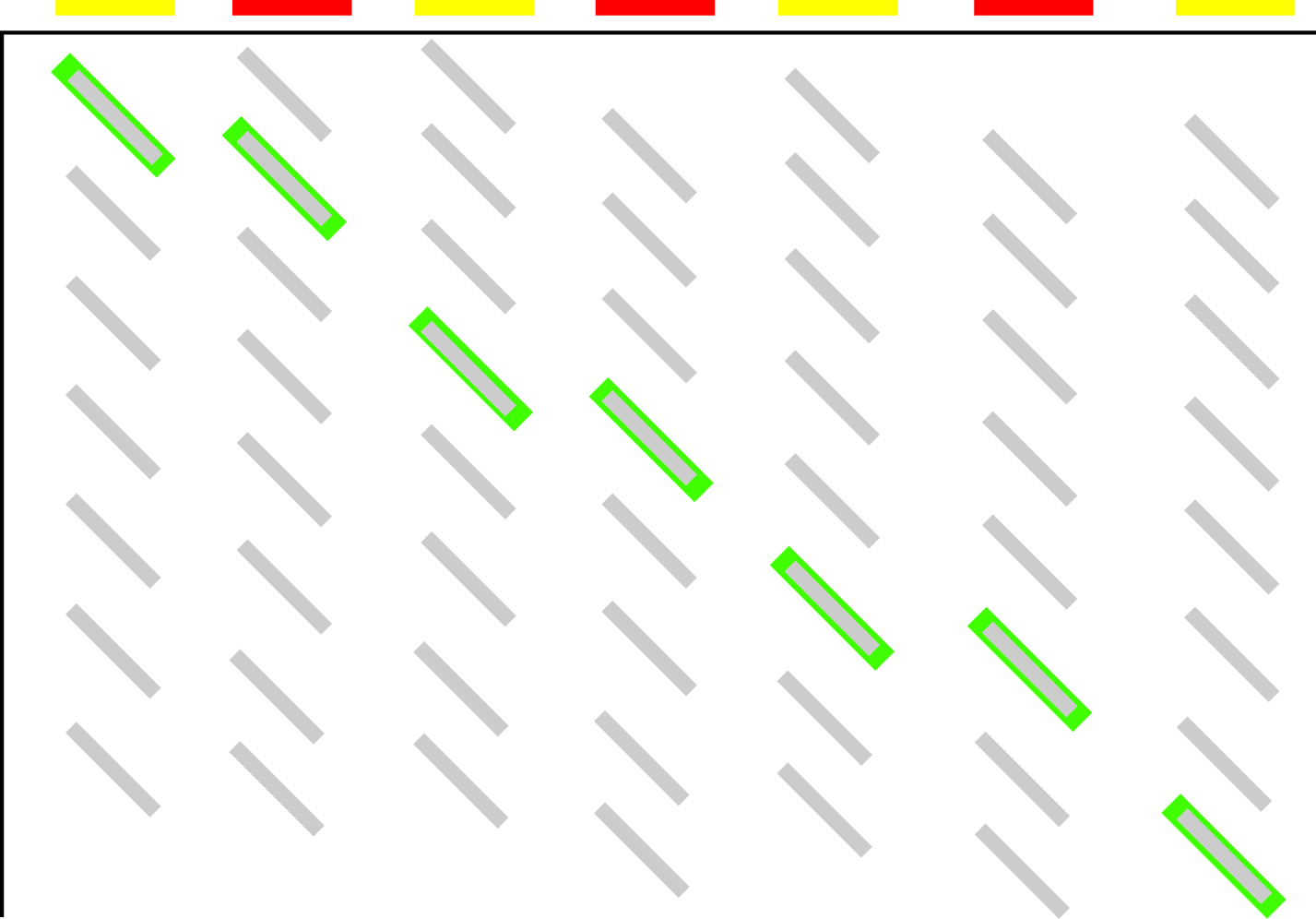


Figure S 1

Figure S1 shows a schematic of a dynamic programming (DP) matrix for a hypothetical alignment of a query protein to a TIM barrel protein. For clarity, the sequence of the query is not shown and only a subset of the full complement of SSE’s in the template is shown here. Secondary structure elements (SSEs) in the template are identified (yellow line=beta strand, red line=alpha helix) and the matrix is divided into columns based on the SSEs. Diagonals in the matrix contained entirely within SSEs (“fragments”) are shown as grey lines with correctly aligned fragments highlighted in green (the correct alignment is the structure based sequence alignment).


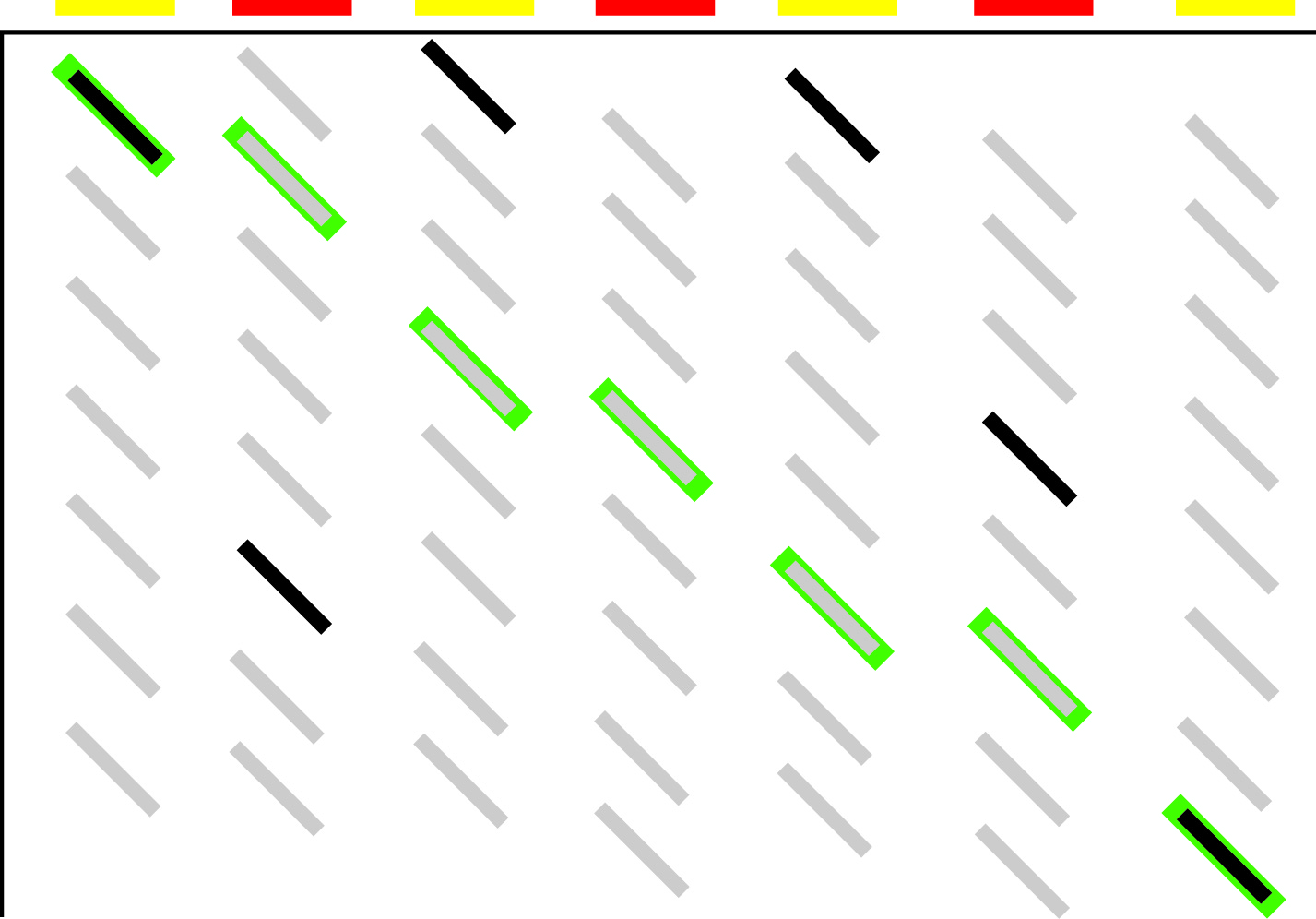


Figure S 2

Every fragment is assigned a score that is the sum of the residue-residue similarity scores in the diagonal. To begin the alignment process, the highest scoring fragment in each column is added to a set of “primary” (Figure S2, black lines). Usually, some primary fragments are correct (e.g., those in the first and last SSE in this example) and some are incorrect. In this example, some columns do not have any primary fragments since they are not in the region of the DP matrix represented here. This situation is typical and we find that as long as some primary fragments are correct, the remainder of the correct alignment can be generated based on a combination of sequence similarity and structural considerations. This is accomplished by identifying “secondary” fragments to connect primary fragments that are on non-consecutive SSE’s.


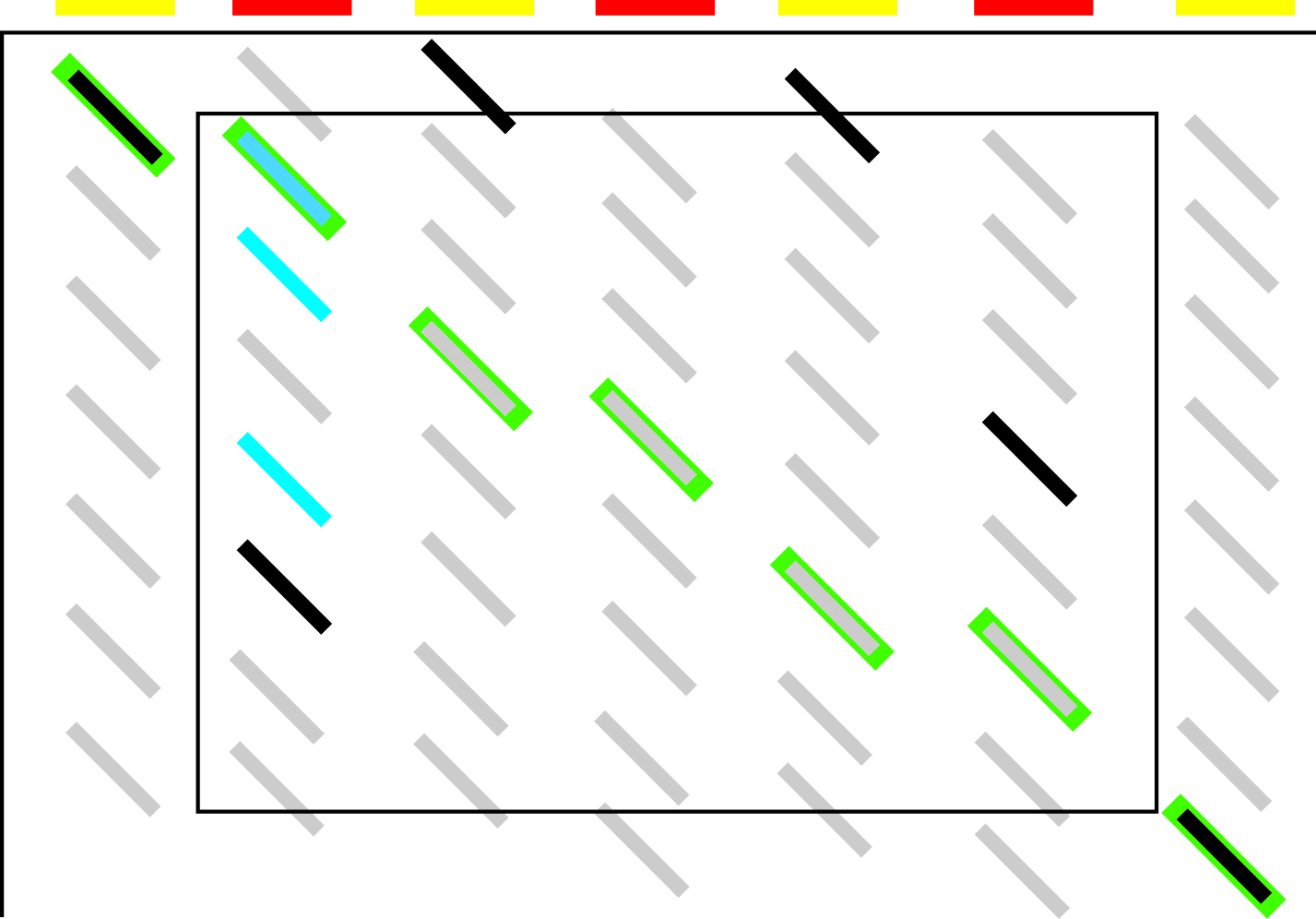


Figure S 3

For example, three secondary fragments were identified to follow the first correct primary fragment (Figure S3, indicated by blue lines). These are chosen from within the region of the DP matrix defined by the first and last primary fragments (black square). The first secondary fragment was chosen based on its adjacency to the black primary fragment and correctly samples a deletion and thus lies on the correct alignment. The second examines the gapless case and stays on the same diagonal to the primary fragment. The third secondary fragment samples the possibility of a large insertion as it lies far from the original diagonal. This fragment was chosen because of its high similarity score. This process of sampling a range of possible insertions and deletions is a core feature of the S4 algorithm. This process continues recursively in successively smaller regions between the primary fragments. Figure S4 shows the remaining secondary fragments as blue lines lying along the correct alignment (green), as well as all the other secondary fragments that were found in order to connect the pair of primary fragments.


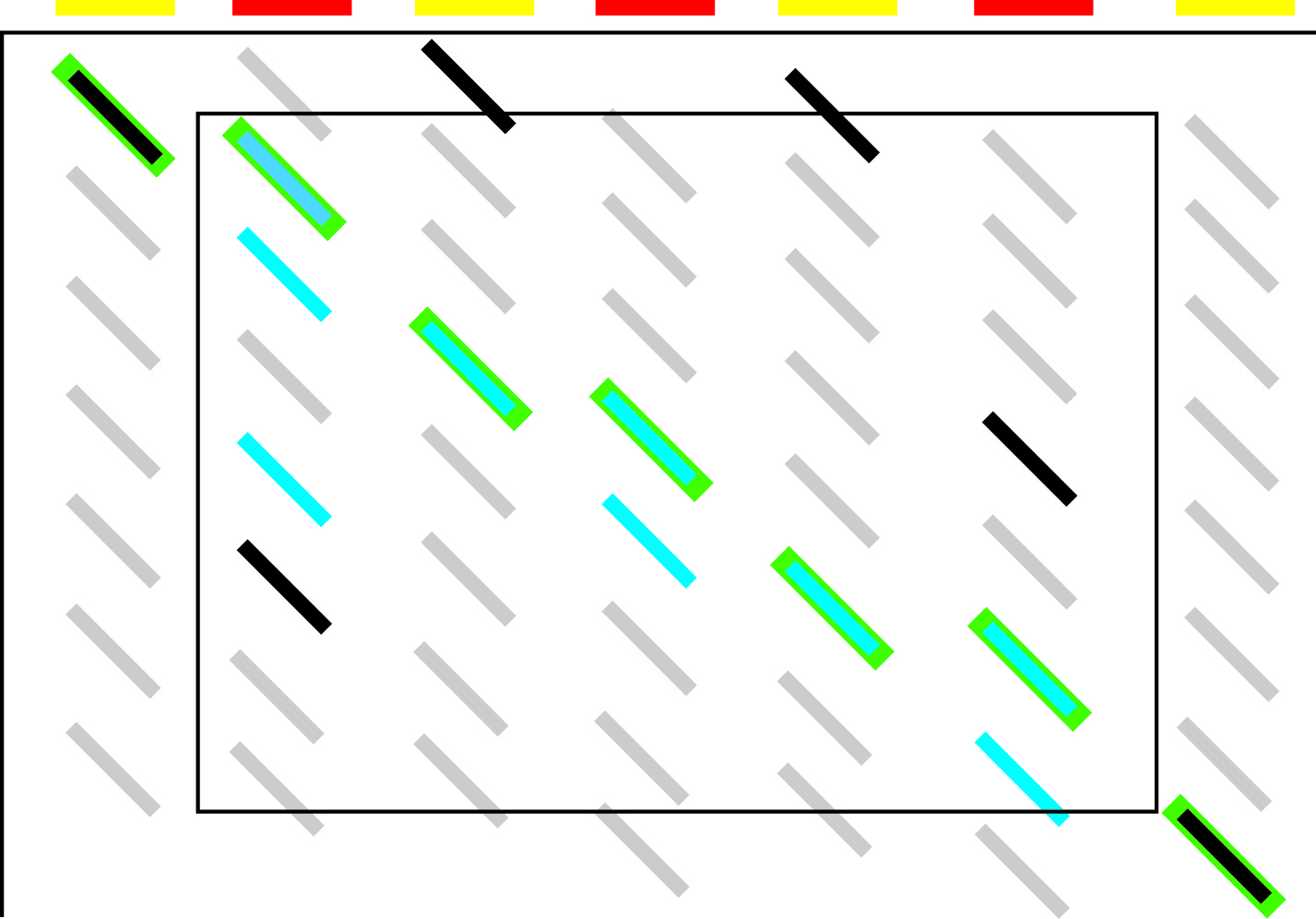


Figure S 4

All combinations of primary fragments and the secondary fragments that connect them are then enumerated (called “fragment alignments”), and assessed by S4’s alignment rules. One can visualize a fragment alignment as a list of primary and secondary fragments ordered from N to C-terminal. Each fragment alignment (i.e., list) defines a narrow region in the matrix. We apply standard dynamic programming constrained to this region to generate a complete alignment of the query to the template.

A concrete example illustrates some of the features of S4 that lead to accurate alignments. Figure S5 shows another alignment of TIM barrels similar to that described above but with specific sequence details. The top five lines in the figure show the index of the template SSEs, the SSE composition of the template and query, the sequence of the template and the correct (structure-based) alignment of the query to that section of the template. The ‘Fragments’ line shows the primary and secondary fragments selected by S4 that led to the best alignment returned out of 1000. The number following each fragment is its z-score, which describes the sequence similarity of the fragment relative to other fragments in the same column. The figure shows only a subregion of the template and query sequences (corresponding to SSEs 4-10, 15-16 of the template), with a large portion of the alignment between SSEs 10 and 15 removed.

Two primary fragments shown in SSE’s 4 and 10 that are part of the correct alignment were chosen in this region. The high sequence similarity for these two fragments (relative to other fragments in their columns) is indicated by their high z-scores (3.9 and 5.8). The other primary fragments in SSEs 5-9 are far from the correct alignment, even though they are the highest scoring fragments in their respective template SSEs. As discussed above, this is often true when the template and query share only remote similarity, but the presence of at least some high-identity fragment is usually sufficient to generate an accurate alignment. The secondary fragments that led to the correct S4 alignment are shown under SSEs 5-9. These fragments were chosen due to their relatively high scores or adjacency to primary fragments.

All combinations of primary fragments and the secondary fragments that connect them are then enumerated. After eliminating many fragment alignments for redundancy and low statistical scores, the algorithm searches for the best full alignment to represent each remaining fragment alignment. A bounded region is drawn narrowly around the fragments (as shown in Figure 1, main text) and the Waterman alternative alignment technique is used to find a set of full alignments. These are evaluated as pseudomodels by DFIRE and the best/lowest-energy alignment is selected. As we can see in the ‘Query S4’ line of Figure S5, which shows the final S4 alignment that resulted from the fragments above it, this step not only fills in the gaps in loop regions, but also often improves the alignment to template SSEs. Of the three fragments that were shifted from their correct positions (SSEs 10, 15 and 16), two of these (15 and 16) were corrected using this procecedure. It should also be noted that this search of a narrow region of alignment space did not disturb the correct alignment of the other six fragments shown. This process continues until the top N alignments, ranked by their original S4 scores calculated in Step 3, are determined.

**Figure S5. Case study illustrating the alignment of a query sequence (deoxyribose-phosphate aldolase) to a template (dihydrodipicolinate synthase, PDB code 2r8w).**


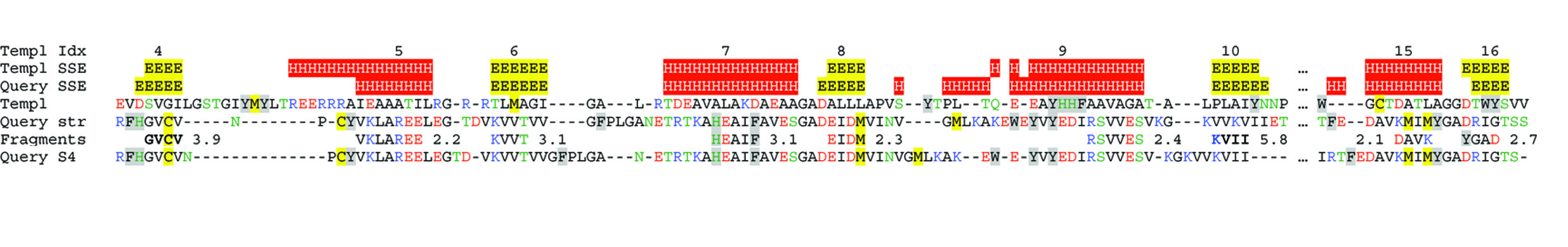


**Figure S6.  The** **constrained version of the Waterman algorithm for making alternative alignments**. During the traceback, the constrained version of the Waterman algorithm can only branch when passing from an SSE region to a loop region, or vice versa.  Here we show three possible alternative alignments generated by the constrained approach: a diagonal ungapped alignment, a branch when transitioning from a helix to a loop, and a branch transitioning from the loop into a strand The primary purpose of only allowing branching at these locations is to eliminate alternative alignments with variations in loop regions that are not meaningfully different (i.e., they produce largely equivalent 3D models).


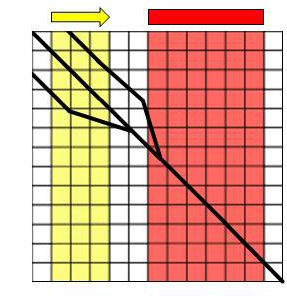

Supplement: Text S1 — Supplemental information for “Using structure to explore the sequence alignment space of remote homologs”. (DOC) [file pcbi.1002175.s001.doc]
